# TIMP2 rs2277698 polymorphism associated with adverse IVF outcomes in Han Chinese women

**DOI:** 10.3389/fendo.2025.1542534

**Published:** 2025-03-13

**Authors:** Chun-I. Lee, Yu-Jen Lee, Tsung-Hsien Lee, Chi-Ying Lee, Hui-Mei Tsao, En-Hui Cheng, Chun-Chia Huang, Shun-Fa Yang, Maw-Sheng Lee

**Affiliations:** ^1^ Division of Infertility, Lee Women’s Hospital, Taichung, Taiwan; ^2^ Department of Obstetrics and Gynecology, School of Medicine, Chung Shan Medical University, Taichung, Taiwan; ^3^ Department of Obstetrics and Gynecology, Chung-Shan Medical University Hospital, Taichung, Taiwan; ^4^ Genetic Diagnosis Laboratory, Lee Women’s Hospital, Taichung, Taiwan; ^5^ Department of Post-Baccalaureate Medicine, College of Medicine, National Chung Hsing University, Taichung, Taiwan; ^6^ Institute of Medicine, Chung-Shan Medical University, Taichung, Taiwan; ^7^ Institute of Bioinformatics and Structural Biology, National Tsing Hua University, Hsinchu, Taiwan

**Keywords:** TIMP2, rs2277698, single nucleotides polymorphisms, clinical outcomes, *in vitro* fertilization

## Abstract

**Background:**

Matrix metalloproteinases (MMPs) and tissue inhibitors of metalloproteinases (TIMPs) are critical regulators of extracellular matrix (ECM) proteolysis and play a pivotal role in trophoblast invasion during embryo implantation. This study aimed to investigate the effects of single-nucleotide polymorphisms (SNPs) in MMP and TIMP genes on clinical outcomes in women undergoing *in vitro* fertilization (IVF).

**Methods:**

This retroprospective study included 1014 women undergoing their first fresh IVF cycle without donor eggs at Lee Women’s Hospital between January 2014 and December 2015. Peripheral blood samples were collected from all participants for DNA extraction and SNP genotyping using real-time polymerase chain reaction. The study focused on three SNPs: TIMP1 (rs4898 C/T), TIMP2 (rs2277698 C/T), and MMP2 (rs243865 C/T). Associations between these SNPs and IVF outcomes, including clinical pregnancy, embryo implantation, abortion, and live birth rates, were analyzed.

**Results:**

Among 560 patients analyzed, no significant differences were observed in baseline characteristics between the live birth and non-live birth groups. However, the minor alleles (CT+TT) of MMP2 (rs243865) and TIMP2 (rs2277698) were significantly more frequent in the non-live birth group (MMP2: 24.4% vs. 17.7%, p = 0.044; TIMP2: 48.1% vs. 34.4%, *p* = 0.001). In contrast, no significant differences in the genotype distribution of TIMP1 (rs4898) were noted between the groups. Logistic regression analysis identified the minor T allele of TIMP2 as a significant predictor of non-live birth (adjusted odds ratio: 1.725; 95% CI: 1.217–2.445; *p* = 0.002). Combined genotypes of MMP2/TIMP2, such as CC/CT+TT and CT+TT/CT+TT, were associated with an increased risk of non-live birth, even after adjusting for covariates.

**Conclusions:**

The study demonstrates that the minor T allele of TIMP2 (rs2277698 C/T) is associated with poor IVF outcomes, particularly non-live birth. This finding highlights the potential role of genetic variations in TIMP2 in influencing clinical outcomes of IVF. Further research is warranted to elucidate the underlying mechanisms in larger and more diverse populations.

## 1 Introduction

Successful pregnancy relies on the proper development and implantation of the embryo. Embryo implantation involves apposition, adhesion, and invasion into the maternal endometrial matrix (1, 2). Extensive degradation and remodeling of the endometrial extracellular matrix (ECM) are crucial for this process, regulated by interactions between ECM components and the matrix metalloproteinase (MMP) family (3). Recent studies continue to highlight the essential role of ECM and its regulators in early pregnancy, with particular focus on novel therapeutic targets for implantation issues (4).

The MMP family consists of structurally related zinc-dependent endopeptidases that degrade various ECM proteins, thereby participating in ECM remodeling (5). These enzymes are involved in multiple physiological processes, including embryo implantation, trophoblast invasion and migration, and endometrial decidualization (6). MMP2, secreted by the embryo during the blastocyst stage, serves as the primary gelatinase in early pregnancy (6 to 8 weeks) and is a key regulator of embryonic invasion. The balance between MMP2 and tissue inhibitors of metalloproteinases (TIMPs) is essential for maintaining normal pregnancy (7, 8).

The MMP2/TIMP2 complex plays a critical role in endometrial remodeling during early pregnancy. Elevated levels of MMP2 and TIMP2 have been observed in patients with spontaneous abortion (9). Singh et al. demonstrated that dysregulation of MMP2 can result in excessive degradation of the endometrial ECM, leading to recurrent spontaneous abortion (RSA) (10). These findings highlight the importance of maintaining the balance between MMP and TIMP expression levels for successful embryo implantation, as an imbalance may contribute to implantation failure (8, 11). A meta-analysis investigating the association between MMP gene polymorphisms and RSA identified a significant correlation between the MMP2 (-735C>T) T allele and RSA risk (12).

To explore these findings further, we selected three specific single-nucleotide polymorphisms (SNPs): TIMP1 rs4898, TIMP2 rs2277698, and MMP2 rs243865. TIMP1 rs4898 and TIMP2 rs2277698 are located within the genes and play key roles in regulating MMP activity, which is crucial for endometrial remodeling and embryo implantation. MMP2 rs243865, located in the promoter region, affects MMP2 expression and alters transcription factor binding sites. Previous studies have shown that these SNPs are associated with recurrent spontaneous abortion or implantation failure (10, 12). Based on this evidence, we chose these loci to investigate their potential impact on IVF outcomes.

Most studies exploring the link between MMPs and infertility have focused on women experiencing RSA. This study investigates the effects of single-nucleotide polymorphisms (SNPs) in MMP and TIMP genes on clinical outcomes in women undergoing their first *in vitro* fertilization (IVF) cycle.

## 2 Materials and methods

### 2.1 Study design and cohort

This retrospective study was conducted at Lee Women’s Hospital (Taichung, Taiwan) from January 2014 to December 2015. We included Han Chinese women under 45 years of age who had undergone their first IVF or intracytoplasmic sperm injection (ICSI) cycle with fresh blastocyst transfer during the study period. Women with autoimmune disorders, inflammatory diseases, genetic abnormalities, or other systemic diseases, as well as those who received donated eggs, were excluded. The study was approved by the Institutional Review Board of Chung Shan Medical University Hospital (CS13194).

Our objective was to identify associations of single-nucleotide polymorphisms (SNPs) in the MMP2 rs243865, TIMP1 rs4898, and TIMP2 rs2277698 genes with IVF clinical outcomes. The primary outcomes assessed were clinical pregnancy, implantation, abortion, and live birth rates. Clinical pregnancy was defined as the detection of an intrauterine gestational sac on ultrasonography. The implantation rate was calculated as the number of observed gestational sacs divided by the number of transferred embryos. Abortion was defined as the natural loss of a pregnancy before the 20th gestational week. Live birth was defined as the delivery of a baby showing signs of life.

### 2.2 Stimulation protocol

All patients underwent ovarian stimulation followed by oocyte retrieval using the gonadotropin-releasing hormone (GnRH) antagonist protocol, as previously described (13). The same protocol was applied to all patients to avoid bias. The procedure began with daily injections of 0.5 mg leuprolide acetate (Lupron; Takeda Pharmaceutics, Konstanz, Germany) starting on day 21 of the previous menstrual cycle. On day 2 or 3 of the cycle, recombinant follicle-stimulating hormone (FSH; Gonal-F; Merck-Serono, Darmstadt, Germany) or highly purified FSH (Menopur; Ferring Pharmaceuticals, Kiel, Germany) was administered in flexible daily doses to promote follicular growth. Human chorionic gonadotropin (hCG; 10,000 IU; Profasi, Serono, Norwell, MA, USA) was used to trigger final oocyte maturation. Oocyte retrieval was performed 36 to 38 hours later. Fertilization was achieved through either conventional insemination or ICSI, depending on semen parameters. Fresh blastocyst transfer was performed within the study period.

### 2.3 DNA extraction and genotyping

Venous blood samples were collected on the day of oocyte retrieval for DNA extraction and subsequent genotyping. Genomic DNA was extracted from ethylenediaminetetraacetate (EDTA)-treated venous blood using the QIAamp DNA Blood Mini Kit (Qiagen, Valencia, USA) according to the manufacturer’s instructions (14). The extracted DNA was dissolved in Tris-EDTA buffer (10 mM Tris, 1 mM EDTA; pH 7.8). DNA quality was assessed by measuring optical density at 260 nm. The final DNA solution was stored at −20°C. This DNA served as the template for polymerase chain reaction (PCR). The evaluated SNPs included MMP2 rs243865, TIMP1 rs4898, and TIMP2 rs2277698, using a TaqMan allelic discrimination assay (TaqMan SNP). Allelic discrimination was performed with the ABI StepOne Real-Time PCR System (Applied Biosystems, Foster City, CA, USA) (15). Primer sequences for each genotype are listed in **Table 1**.

**Table 1:** Primer sequences used for assessing the indicated SNPs.

Gene (SNP ID)	Functional Consequence	Region	Context Sequence
MMP2 (rs243865)	-1306C>T	Non-coding (intron)	TCCCCATATTCCCCACCCAGCACTC[C/T] ACCTCTTTAGCTCTTCAGGTCTCAG
TIMP1 (rs4898)	372 T>C silent mutation	Coding (exon)	TCTTGCACATCACTACCTGCAGTTT[C/T] GTGGCTCCCTGGAACAGCCTGAGCT
TIMP2 (rs2277698)	303 G>A silent mutation	Coding (exon)	ATTCCTTCTTTCCTCCAACGTCCAG[C/T] GAGACCCCACACACTGCCGAGGAGG

MMP, matrix metalloproteinase; SNP, single-nucleotide polymorphism; TIMP, tissue inhibitor of metalloproteinase.

### 2.4 Propensity score matching

**Figure 1:**
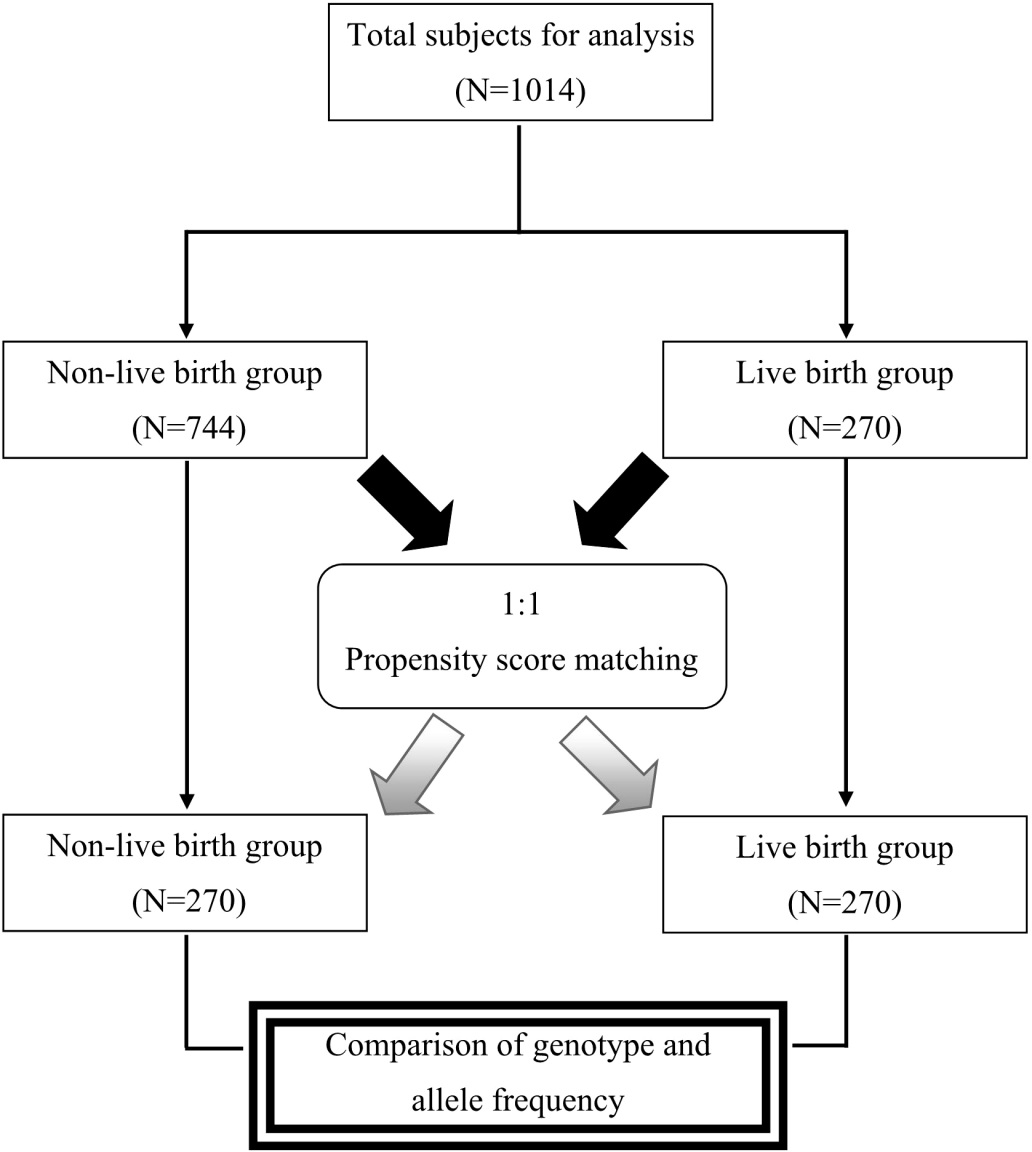
Flowchart depicting group allocation before and after propensity score matching.

To analyze the effects of SNPs on live birth, we performed propensity score matching (PSM) to control for factors influencing live birth. Covariates used for calculating propensity scores included maternal age, duration of infertility, anti-Müllerian hormone (AMH) levels, and endometrial thickness on the day of embryo transfer. We employed the nearest neighbor matching algorithm with caliper matching, setting the standard deviation of the logit of the propensity score at 0.02. We matched 270 women who experienced non-live birth with 270 women who experienced live birth (control group) in a 1:1 ratio. **Figure 1** presents a flowchart illustrating the group allocation.

### 2.5 Statistical analysis

Continuous variables were compared using Student’s t-test and are presented as mean ± SD values. Categorical variables were compared using the chi-square test and are presented as numbers (percentages). Associations between the tested SNPs and live birth were investigated using different genetic models: codominant (AA vs. Aa vs. aa), dominant (AA+Aa vs. aa), and allelic (A vs. a) models, employing the chi-square test. To enhance accuracy, both unadjusted (univariate) and adjusted (multivariate) analyses were performed using logistic regression, conducted through a generalized estimating equation model. Data analysis was performed using SPSS Statistics (version 22.0) for Windows (IBM Corporation, Armonk, NY, USA). A *p*-value of <0.05 was considered statistically significant.

## 3 Result

### 3.1 Baseline characteristics and cohort composition

The patient cohort in this study comprised 1014 women. **Table 2** presents the baseline characteristics of the study groups both before and after propensity score matching (PSM). Initially, we identified 270 women who had experienced live birth and 744 women who had experienced non-live birth. Before PSM, significant differences between the groups were observed in maternal age (*p* < 0.001), duration of infertility (*p* = 0.045), AMH levels (*p* < 0.001), basal FSH levels (*p* = 0.018), and endometrial thickness on the day of embryo transfer (*p* = 0.005). After PSM, these differences were no longer significant, indicating that comparable cohorts had been established.

**Table 2:** Baseline characteristics of the study groups before and after propensity score matching.

Characteristics	Before PSM		*p* value	After PSM		*p* value
	Non-live birth (n=744)	Live birth (n=270)		Non-live birth (n=270)	Live birth (n=270)	
Maternal age (years)	36.2 ± 4.7	33.6 ± 3.7	<0.001**	33.7 ± 4.1	33.6 ± 3.7	0.79
Duration of infertility (years)	3.8 ± 2.9	3.4 ± 2.7	0.045*	3.4 ± 2.5	3.4 ± 2.7	0.942
AMH (ng/mL)	2.81 ± 2.72	3.97 ± 3.23	<0.001**	3.63 ± 2.84	3.97 ± 3.23	0.183
Baseline FSH (mIU/mL)	7.28 ± 4.66	6.44 ± 3.34	0.018*	6.99 ± 3.56	6.44 ± 3.34	0.242
Baseline LH (mIU/mL)	6.39 ± 5.92	6.70 ± 5.03	0.607	6.70 ± 5.57	6.70 ± 5.03	1
Baseline E2 (pg/mL)	49.59 ± 7.17	58.93 ± 10.23	0.168	47.19 ± 7.84	58.93 ± 10.23	0.134
E2 on day of trigger (pg/mL)	1794.16 ± 1333.31	2145.66 ± 1332.36	0.001**	2175.94 ± 1421.98	2145.66 ± 1332.36	0.826
P4 on day of trigger (pg/mL)	1.88 ± 0.69	0.97 ± 0.51	0.085	0.98 ± 0.39	0.97 ± 0.51	0.839
Day of stimulation (days)	14.0 ± 1.7	13.9 ± 1.2	0.104	14.0 ± 1.5	13.9 ± 1.2	0.424
No. of retrieved oocytes (n)	9.4 ± 6.5	11.4 ± 6.8	<0.001**	11.4 ± 6.7	11.4 ± 6.8	0.491
No. of mature oocytes (n)	7.5 ± 6.2	9.8 ± 5.7	<0.001**	9.5 ± 7.2	9.8 ± 5.7	0.543
No. of Day3 embryos (n)	6.8 ± 4.9	7.6 ± 5.0	0.001**	8.4 ± 5.4	7.6 ± 5.0	0.117
Day3 good embryo rate (%)	60.0 ± 24.2	62.1 ± 22.3	0.308	61.3 ± 20.3	62.1 ± 22.3	0.749
Day5 good embryo rate (%)	44.1± 20.5	47.6 ± 21.8	0.51	45.4 ± 23.4	47.6 ± 21.8	0.491
No. of transferred embryos (n)	2.5 ± 0.9	2.6 ± 0.7	0.114	2.6 ± 0.8	2.6 ± 0.7	0.347
Endometrial thickness (mm)#	11.6 ± 1.5	12.0 ± 1.9	0.005**	11.9 ± 1.7	12.0 ± 1.9	0.467

AMH, anti-Müllerian hormone; E2, estradiol; FSH, follicle-stimulating hormone; LH, luteinizing hormone; PSM, propensity score matching.

Data are presented in terms of mean ± SD values. *P* values were calculated using Student’s *t* test. *p < 0.05 and **p < 0.01 indicate statistical significance. #Endometrial thickness on the day of embryo transfer.

### 3.2 Clinical outcomes after propensity score matching

Subsequently, we examined the clinical outcomes of the two groups after PSM, as outlined in **Table 3**. Significant differences were observed between the groups in the rates of clinical pregnancy, implantation, and miscarriage (*p* < 0.001). The non-live birth group exhibited a substantially high miscarriage rate (79.2%), even among individuals who initially achieved pregnancy. Based on these findings, we hypothesized that differences in genotype distribution might influence the clinical outcomes between the two groups.

**Table 3:** Clinical outcomes in the non-live birth and live birth groups after propensity score matching.

	Live birth		
	No (n=270)	Yes (n=270)	*p* value
Clinical pregnancy rate (%)	17.8 (48/270)	100 (270/270)	<0.001*
Implantation rate (%)	7.5 (53/707)	55.9 (386/691)	<0.001*
Abortion rate (%)	79.2 (38/48)	0 (0/270)	<0.001*

Data are presented in terms of number (percentage) values. *P* values were calculated using the chi-square test. *p < 0.001 indicates statistical significance.

### 3.3 Genotype distribution and allele frequencies

The genotype distribution and allele frequencies of MMP and TIMP polymorphisms between the study groups are presented in **Table 4**. For the MMP2 C>T polymorphism (rs243865), the T allele exhibited a higher frequency in the non-live birth group than in the live birth group (13.1% vs. 9.1%; *p* = 0.033). When genotypes carrying the minor allele were combined (CT+TT), the allele frequency was significantly higher in the non-live birth group compared to the live birth group (24.4% vs. 17.7%; *p* = 0.044). Similarly, the T allele of the TIMP2 C>T SNP (rs2277698) displayed a higher frequency in the non-live birth group (27.2% vs. 18.7%; *p* = 0.001). Genotypes carrying the minor alleles exhibited significantly increased frequencies in both codominant and dominant models (CT: 41.9% vs. 31.5%; TT: 6.3% vs. 2.9%; CT+TT: 48.1% vs. 34.4%; *p* = 0.001). However, no significant between-group differences were observed in the allele frequency or genotype distribution of the TIMP1 T>C SNP (rs4898).

**Table 4:** Genotype distribution and allele frequencies of MMP2, TIMP1 and TIMP2 SNP in the non-live birth group and live birth group.

SNP	Model	Genotype	Live birth		*p* value
			No (n=270)	Yes (n=270)	
MMP2 rs243865	Codominant	CC	204 (75.6%)	223 (82.6%)	0.103
		CT	61 (22.6%)	45 (16.7%)	
		TT	5 (1.9%)	2 (0.7%)	
	Dominant	CC	204 (75.6%)	223 (82.6%)	0.044*
		CT+TT	66 (24.4%)	47 (17.7%)	
	Allele	C	469 (86.9%)	491 (90.9%)	0.033*
		T	71 (13.1%)	49 (9.1%)	
TIMP1 rs4898	Codominant	TT	78 (28.9%)	82 (30.4%)	0.609
		TC	140 (51.9%)	129 (47.8%)	
		CC	52 (19.3%)	59 (21.9%)	
	Dominant	TT	78 (28.9%)	82 (30.4%)	0.706
		TC+CC	192 (71.1%)	188 (69.6%)	
	Allele	T	296 (54.8%)	293 (54.3%)	0.855
		C	244 (45.2%)	247 (45.7%)	
TIMP2 rs2277698	Codominant	CC	140 (51.9%)	177 (65.6%)	0.003**
		CT	113 (41.9%)	85 (31.5%)	
		TT	17 (6.3%)	8 (2.9%)	
	Dominant	CC	140 (51.9%)	177 (65.6%)	0.001**
		CT+TT	130 (48.1%)	93 (34.4%)	
	Allele	C	393 (72.8%)	439 (81.3%)	0.001**
		T	147 (27.2%)	101 (18.7%)	

MMP, matrix metalloproteinase; SNP, single-nucleotide polymorphism; TIMP, tissue inhibitor of metalloproteinase.

Data are presented in terms of number (percentage) values. *P* values were calculated using the chi-square test. *p < 0.05 and **p < 0.01 indicate statistical significance.

### 3.4 Logistic regression analysis of predictors for non-live birth

**Table 5** presents the odds ratios (ORs) for the association between gene polymorphisms and non-live birth. A logistic regression analysis was conducted to investigate genotypes as predictors of non-live birth among women undergoing IVF. The univariate analysis revealed that genotypes containing the minor allele (CT+TT) of MMP2 or TIMP2 were significant predictors of non-live birth (unadjusted OR for MMP2: 1.535, *p* = 0.045; unadjusted OR for TIMP2: 1.767, *p* = 0.001). After adjusting for covariates, only the genotype containing the minor allele of TIMP2 remained a significant predictor (adjusted OR: 1.725; 95% CI: 1.217 to 2.445; *p* = 0.002).

**Table 5:** Odds ratios for association between gene polymorphisms and non-live birth (dominant model).

Genotype	Univariate Regression		Multivariate Regression	
	OR (95% CI)	*p* value	OR (95% CI)	*p* value
MMP2 rs243865				
CC	Ref		Ref	
CT+TT	1.535 (1.009-2.335)	0.045*	1.462 (0.955-2.240)	0.081
TIMP2 rs2277698				
CC	Ref		Ref	
CT+TT	1.767 (1.250-2.449)	0.001**	1.725 (1.217-2.445)	0.002**
Combined genotype MMP2/TIMP2				
CC/CC	Ref		Ref	
CC/CT+TT	1.687 (1.140-2.496)	0.009**	1.681 (1.134-2.491)	0.010*
CT+TT/CC	1.390 (0.783-2.469)	0.261	1.383 (0.076-2.466)	0.271
CT+TT/CT+TT	2.614 (1.427-4.788)	0.002**	2.627 (1.431-4.823)	0.002**

MMP, matrix metalloproteinase; OR, odds ratio; TIMP, tissue inhibitor of metalloproteinase.

A generalized estimating equation model was used for logistic regression. The multivariate model was adjusted for age, infertility duration, transferred embryo count, endometrial thickness on the day of embryo transfer, and genotype. *p < 0.05 and **p < 0.01 indicate statistical significance.

### 3.5 Combined analysis of MMP2 and TIMP2 genotypes

A combined analysis was performed using the dominant models for MMP2 (CC) and TIMP2 (CT+TT). Compared with the reference genotype, the combined MMP2/TIMP2 genotypes CC/CT+TT and CT+TT/CT+TT were associated with 1.687- and 2.614-fold increases in the risk of non-live birth (unadjusted OR for CC/CT+TT: 1.687, *p* = 0.009; unadjusted OR for CT+TT/CT+TT: 2.614, *p* = 0.002). After adjusting for covariates, these combined genotypes remained significant predictors (adjusted OR for CC/CT+TT: 1.681, *p* = 0.010; adjusted OR for CT+TT/CT+TT: 2.627, *p* = 0.002). However, no significant association was observed between the CT+TT/CC genotype of MMP2/TIMP2 and non-live birth.

These findings indicate that the TIMP2 genotype containing the minor allele (T allele) is a key predictor of the risk of non-live birth among women undergoing IVF.

## 4 Discussion

MMP2 rs243865 polymorphism is a point mutation located in the promoter region (–1306), where a C→T transition disrupts the binding of specificity protein 1 (SP1), significantly reducing promoter activity and downregulating MMP2 expression (16–18). Studies have demonstrated that MMP2 expression in carriers of the variant genotypes (CT+TT) is significantly lower than in those with the wild-type genotype (CC) (19). In Chinese women, the MMP2 rs243865 T allele is closely associated with the risk of recurrent spontaneous abortion (RSA) (20). Our study further supports these findings, revealing that the clinical pregnancy and implantation rates in the non-live birth group were significantly lower than those in the live birth group, while the miscarriage rate was notably higher. Moreover, the distribution of the variant genotypes (CT+TT) differed significantly between groups, suggesting that the MMP2 rs243865 T allele may contribute to adverse pregnancy outcomes by influencing extracellular matrix (ECM) regulation.

As the primary endogenous regulator of MMP2, TIMP2 maintains ECM balance by forming TIMP2/MMP2 complexes (21). TIMP2 exerts a concentration-dependent dual effect on MMP2 activity: low concentrations enhance MMP2 activity, whereas high concentrations inhibit it (22–24). TIMP2 rs2277698 polymorphism involves a C→T transition at position 303, resulting in a synonymous mutation (Ser101). Although the precise impact of this polymorphism on TIMP2 expression remains unclear, bioinformatic analyses suggest its potential role in splicing regulation and alterations in transcriptional activity. Moreover, its strong linkage disequilibrium with other SNPs, such as rs9889410 and rs11654470, may further modulate TIMP2 expression (25–27), emphasizing the complexity of genetic variations in the regulation of the extracellular matrix (ECM).

Evidence from studies on recurrent pregnancy loss (RPL) indicates that TIMP2 is overexpressed in both murine models and women with a history of RPL, suggesting its potential as a predictive marker for RPL (6, 28, 29). Excessive TIMP2 expression disrupts the fine-tuned MMP2/TIMP2 balance, leading to abnormal ECM degradation in the endometrium, which interferes with embryo implantation and normal placental development, potentially causing spontaneous miscarriage (6, 9). In our study, the proportion of carriers of the TIMP2 rs2277698 T allele genotype was significantly higher in the non-live birth group compared to the live birth group. This association was validated under codominant and dominant genetic models. Logistic regression analysis further identified the T allele as an independent predictor of non-live birth, underscoring its potential impact on pregnancy outcomes.

While our study establishes a significant association between TIMP2 rs2277698 polymorphism and adverse pregnancy outcomes, certain limitations must be acknowledged. The primary limitation is the lack of functional experiments to elucidate how the TIMP2 rs2277698 polymorphism influences gene expression and its downstream effects on IVF outcomes. Previous studies have suggested that synonymous mutations, such as rs2277698, may affect mRNA splicing efficiency and stability, thereby altering protein expression (25, 27). To address this gap, future studies should include gene expression analyses using qRT-PCR and Western blot to quantify TIMP2 levels in carriers of different genotypes. Furthermore, luciferase reporter assays could provide mechanistic insights into the impact of this polymorphism on transcriptional activity.

Another limitation is the absence of long-term follow-up data to assess the effects of identified SNPs on offspring health and developmental milestones. Existing evidence suggests that dysregulation of the MMP2/TIMP2 axis during pregnancy may impair placental function, thereby affecting fetal development (28, 29). Longitudinal studies linking maternal genotypes to offspring outcomes, such as growth, cognitive development, and metabolic health, are critical for understanding the broader clinical implications of these genetic associations.

Furthermore, our study population was limited to Han Chinese women undergoing their first IVF cycle, which may introduce selection bias and restrict the generalizability of the findings to other populations and natural pregnancies. To mitigate this limitation, large-scale studies in diverse populations are warranted to validate and extend our findings.

Lastly, the therapeutic potential of targeting the TIMP2/MMP2 balance remains an area for exploration. Preclinical studies suggest that small-molecule inhibitors or recombinant TIMP2 proteins may restore ECM balance under pathological conditions (21, 22). Additionally, antioxidant supplementation, such as resveratrol, has shown promise in modulating MMP activity (24). Experimental studies investigating these therapeutic approaches in IVF patients carrying the TIMP2 rs2277698 T allele could provide novel strategies to improve reproductive outcomes.

In conclusion, our study highlights the association between TIMP2 rs2277698 polymorphism and adverse clinical outcomes in IVF. These findings emphasize the critical role of maintaining ECM remodeling balance and advocate for further research into functional mechanisms, long-term offspring outcomes, and therapeutic interventions to enhance reproductive success.

## Ethics committee approval

All elements of this study involving human participants were reviewed and approved by the Institutional Review Board of Chung Shan Medical University, Taichung, Taiwan (CS13194). The ethics committee waived the requirement for written informed consent for participation.

## Data Availability

The raw data supporting the conclusions of this article will be made available by the authors without undue reservation.
